# Biotransformation of Oleanane and Ursane Triterpenic Acids

**DOI:** 10.3390/molecules25235526

**Published:** 2020-11-25

**Authors:** Natalia A. Luchnikova, Victoria V. Grishko, Irina B. Ivshina

**Affiliations:** 1 Institute of Ecology and Genetics of Microorganisms, Perm Federal Research Center, Ural Branch of the Russian Academy of Sciences, 614081 Perm, Russia; luchnikova.n@mail.ru; 2 Department of Microbiology and Immunology, Perm State National Research University, 614990 Perm, Russia; 3 Institute of Technical Chemistry, Perm Federal Research Center, Ural Branch of the Russian Academy of Sciences, 614013 Perm, Russia; grishvic@gmail.com

**Keywords:** biological activity, biotransformation, glycyrrhetinic acid, oleanolic acid, ursolic acid

## Abstract

Oleanane and ursane pentacyclic triterpenoids are secondary metabolites of plants found in various climatic zones and regions. This group of compounds is highly attractive due to their diverse biological properties and possible use as intermediates in the synthesis of new pharmacologically promising substances. By now, their antiviral, anti-inflammatory, antimicrobial, antitumor, and other activities have been confirmed. In the last decade, methods of microbial synthesis of these compounds and their further biotransformation using microorganisms are gaining much popularity. The present review provides clear evidence that industrial microbiology can be a promising way to obtain valuable pharmacologically active compounds in environmentally friendly conditions without processing huge amounts of plant biomass and using hazardous and expensive chemicals. This review summarizes data on distribution, microbial synthesis, and biological activities of native oleanane and ursane triterpenoids. Much emphasis is put on the processes of microbial transformation of selected oleanane and ursane pentacyclic triterpenoids and on the bioactivity assessment of the obtained derivatives.

## 1. Introduction

Drugs derived from secondary plant metabolites make up about 25% of the global pharmaceutical market [1]. Secondary metabolites of plants are several groups of compounds; the most numerous (about 25,000 representatives) and diverse group is terpenic hydrocarbons and their oxygen-containing derivatives (terpenoids). Depending on the number of isoprene units (C_5_H_8_) in their structure, they contain a certain number of carbon atoms and are classified into mono-(C_10_), sesqui-(C_15_), di-(C_20_), triterpenoids (C_30_), etc.

Naturally occurring triterpenoids are represented by more than 100 various types of skeletons [2]. Native triterpenoids, in particular, oleanane and ursane representatives, are of interest for researchers due to their availability and multiple biological activities, including antimicrobial, anti-inflammatory, antitumor, cytotoxic, hepatoprotective, and other activities [3,4,5,6,7]. Triterpenic molecules, however, are highly hydrophobic which significantly limits their use as effective pharmacological agents. At present, one of the most common ways to increase the effectiveness and bioavailability of triterpenoids is by chemical modification. This usually requires high temperature and pH, use of expensive reagents, and introduction of protective groups of molecule reactive centers [8,9,10,11]. An alternative way to obtain valuable derivatives is by biotransformation under normal and environmentally friendly conditions employing the catalytic activity of microorganisms with high regio- and stereoselectivity in one technological stage. Furthermore, microbial conversion ensures specific modifications of triterpenic molecule sites that are either not modified or poorly modified by synthetic transformations [12]. Note that, among the known microbial biocatalysts, members of mycelial fungi are the most studied [13,14,15] whereas bacterial catalysts are only represented by a few gram-positive species [16,17,18,19,20]. The first papers on microbial transformation of triterpenoids were published in the 1960s [21]. The earliest information related to bioconversion processes of oleanane derivatives catalyzed by fungi, such as *Curvularia lunata* ATCC 13432, *Trichotecium roseum* ATCC 8685, *Cunninghamella* sp. ATCC 3229, *Mucor griseo-cyanus* ATCC 1207-a, *Helicostylum piriforme* ATCC 8992, *Fusarium lini*, and *Cunninghamella blakesleana* dates back to about the same time [22,23,24,25]. The data on bacterial transformation of oleanane triterpenoids by *Streptomyces* sp. G-20 and *Chainia antibiotica* IFO 12,246 were reported in the second half of the 1980s [26,27]. As for microbial transformations of ursane pentacyclic triterpenoids by both fungal (*Mucor plumbeus* ATCC 4740 [28]) and bacterial (*Nocardia* sp. NRRL 5646 [29]) strains, those studies were initiated only in the 2000s. Henceforth, the interest in the topic discussed has been increasing and the Active Triterpenoid Biocatalysts List has been expanded every year, as is the number of various bioactive triterpenic derivatives formed via biotransformations [30,31,32].

Now, preparation of biologically active compounds based on pentacyclic triterpenoids is an actual research discussed in plenty of experimental and review publications [8,12,32,33,34]. However, the reviews are overwhelmingly focused on chemical transformations or describe specific types of biological activities of triterpenoids. Less frequently, they deal with biological transformations. The latest of the few reviews on microbial transformations of pentacyclic triterpenoids include literature data up to 2016 [12,32,33,35]. Our review summarizes the data from 2013 to the present on distribution, microbial biosynthesis, biological activity, and mainly biotransformation of oleanane and ursane pentacyclic triterpenoids to obtain promising biologically active compounds or intermediates of their synthesis.

## 2. Distribution in Nature

**Figure 1:**
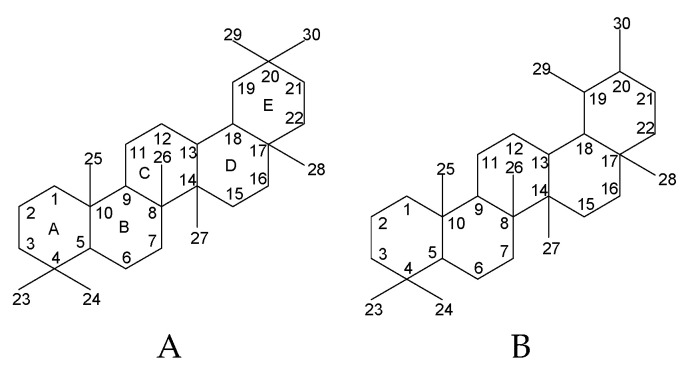
Representative skeletons of oleanane (**A**) and ursane (**B**) pentacyclic triterpenoids.

Plant pentacyclic triterpenoids are represented by more than two dozen structural types; the most common are oleanane and ursane ones (Figure 1). In free forms, triterpenoids are nonvolatile lipophilic substances that are soluble in organic solvents and insoluble in water. The most available oleanane triterpenoids are oleanolic acid **1** (OA, 3β-hydroxy-olean-12-en-28-oic acid, C_30_H_48_O_3_, CAS 508-02-1) and glycyrrhetinic acid **2** (GA, 3β-hydroxy-11-oxo-18β-olean-12-en-30-oic acid, C_30_H_46_O_4_, CAS 471-53-4), and ursolic acid **3** (UA, 3β-hydroxy-urs-12-en-28-oic acid, C_30_H_48_O_3_, CAS 77-52-1) is the most available ursane triterpenoid. Biosynthesis of oleanane and ursane pentacyclic triterpenoids in plants occurs by conversion of the acyclic triterpene squalene (**4**) to 2,3-oxidosqualene (**5**) and its further cyclization by specific enzyme complexes (oxidosqualene cyclase) via β-amyrin (**6**) or α-amyrin (**7**), respectively [36,37]. The carbon skeletons of these triterpenoids consist of five condensed cyclohexane rings. What differentiates them is one methyl group (CH_3_-29) located in oleanane and ursane derivatives at C20 and C19 of the E ring, respectively.


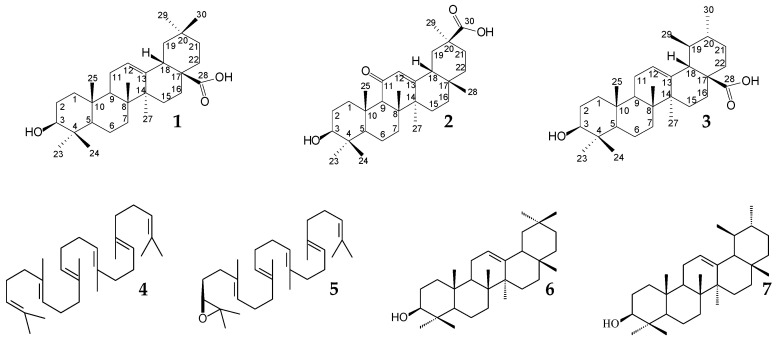


Representatives of various higher plant families are active producers of both oleanane and ursane triterpenoids (Table 1). Frequently, OA and UA are simultaneously detected in the same plant sources. OA and UA contents in *Meconopsis henrici*, *Dracocephalum tanguticum*, *Comastoma pulmonaria*, *Corydalis impatiens*, and *Swertia racemosa*—traditionally used in Chinese medicine—can reach 0.96 ± 0.01 mg/g and 0.64 ± 0.01 mg/g dry weight, respectively [38]. Flowers and leaves of the shrubs *Ocimum tenuiflorum* and *Syzygium aromaticum* and the herbs *Origanum vulgare*, *Rosmarus officinalis*, and *Salvia officinalis* used as condiments contain up to 15.3 mg/g OA and up to 26.2 mg/g UA (wet weight) [39]. The main source of OA is considered to be the fruits and leaves of *Olea europaea*. The acid content in olive leaves can reach 27.16 mg/g wet weight and 25.09 ± 0.72 mg/g dry weight [39,40]. GA is commonly extracted from herbaceous plants of the genus *Glycyrrhiza* [41,42,43]; the acid content in their roots can reach 10.2 ± 1.7 mg/g wet weight [44].

The amount of pentacyclic triterpenoids in plants is not constant and can significantly vary depending on the activity of enzyme systems and external factors [45]. Thus, the fruits and leaves of olive (*Olea europaea*) of various varieties contained OA from 0.4 ± 0.1 mg/g to 0.81 ± 0.16 mg/g dry weight and from 29.2 ± 1.8 mg/g to 34.5 ± 3.1 mg/g dry weight, respectively [46,47]. The OA content decreased by 70‒80% during olive fruit ripening [40]. The same tendency was observed when grapes (*Vitis vinifera*) ripen [48]. Changes in pentacyclic triterpenoid concentrations in plant sources may be related to specific climate, season, landscape, and cultivation strategies [47].

## 3. Biosynthesis of Pentacyclic Triterpenic Acids Using Microorganisms

**Scheme 1:**
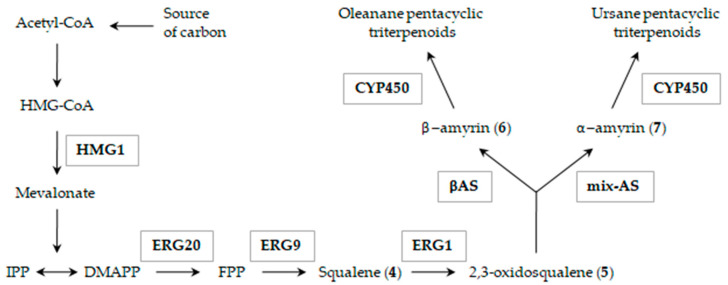
Biosynthesis of oleanane and ursane triterpenoids in engineered yeasts. Enzymes (in frames): HMG1, 3-hydroxy-3-methylglutaryl-CoA reductase; ERG20, farnesyl phosphate synthase; ERG9, squalene synthase; ERG1, squalene epoxidase; βAS, β-amyrin synthase; mix-AS, mixed amyrin synthase; CYP450, cytochrome P450. Metabolites: IPP, isopentenyl diphosphate; DMAPP, dimethylallyl diphosphate; and FPP, farnesyl diphosphate. Modified from Lu et al., 2018 and Zhao et al., 2018 [64,65].

Today, pentacyclic triterpenoids and their natural derivatives are mainly obtained by extraction from plant sources. However, the extraction and separation of these compounds (often with organic solvents) are extremely labor-intensive, and energy- and time-consuming. Besides, most of pentacyclic triterpenoids are found in relatively low concentrations in plants, entailing the use of huge amounts of plant raw materials and the formation of waste biomass in large volumes [62]. An alternative source of pentacyclic triterpenoids seems to be highly efficient cell factories, increasingly popular in the last decade. They allow to obtain valuable biologically active compounds of plant origin in environmentally friendly conditions using available compounds as the sole source of carbon (glucose, galactose, and ethanol) [63]. Cell factories are usually yeast cells—natural catalysts of the mevalonate (MVA) pathway—with plant genes responsible for pentacyclic triterpenoid synthesis introduced into their genome. The MVA pathway includes formation of mevalonate involving 3-hydroxy-3-methylglutaryl-CoA reductases (HMG1). The mevalonate formed is further transformed into isopentenyl diphosphate and dimethylallyl diphosphate, being converted to farnesyl diphosphate by a farnesyl phosphate synthase (ERG20). This pathway provides natural synthesis of squalene (**4**)—a common precursor of triterpenoids—by the squalene synthase (ERG9) based on two molecules of farnesyl diphosphate and its further transformation into 2,3-oxidosqualene (**5**) by squalene epoxidase (ERG1) [64]. Subsequent synthesis of pentacyclic triterpenoids involves plant genes encoding amyrin synthase, CYP450, and CYP450 reductase (Scheme 1).

Genes encoding β-amyrin synthase (βAS) that catalyze the formation of β-amyrin (**6**)—a precursor of oleanane pentacyclic triterpenoids—from 2,3-oxidosqualene (**5**) were isolated from the genomes of *Glycyrrhiza glabra*, *Panax ginseng*, *Catharanthus roseus*, *Lotus japonicus*, *Artemisia annua*, *Chenopodium quinoa*, and others [65,66,67,68,69]. Because no enzymes were found to synthesize exclusively α-amyrin (**7**), a precursor of ursane pentacyclic triterpenoids, this reaction involves mixed amyrin synthases (mix-AS) that catalyze the formation of both α- and β-amyrin from *Eriobotrya japonica* and *C*. *roseus* [64,70]. *Medicago truncatula* is most often used to search for genes encoding CYP450 enzymes that catalyze subsequent conversion of α- and β-amyrin [64,65,66,67,69,71]. Less frequently, *Phaseolus vulgaris* [72], *Bupleurum falcatum* [71], *G*. *uralensis* [62], *C*. *roseus* [64], *Crataegus pinnatifida* [70], *Solanum lycoperum*, *P*. *ginseng* [68], and others are used to search for these genes. Native microbial cytochrome P450 reductases are often unable to transfer electrons to foreign CYP450s, as required for catalysis, and the source of additional CYP450 reductases (CPR and ATR) is usually *Arabidopsis thaliana* [62,64,65,66,69,71]. In a few studies, CYP450 reductases were obtained from *M*. *truncatula* [65], *L*. *japonicus* [67], *G*. *uralensis* [62], and *V*. *vinifera* [70]. Various approaches, including modification, overexpression, or inactivation of microorganisms’ own genes; insertion of plant genes in the yeast genome by various techniques; as well as combinatorial biosynthesis, are used to enhance microbial biosynthesis and to achieve an increased yield of pentacyclic triterpenoids (Table 2).

Overexpression of genes *ERG1*, *ERG9*, *ERG20*, and, more frequently, *tHMG1* (HMG1 with truncated *N*-terminal 511 amino acids) involved in the natural microbial synthesis of 2,3-oxidosqualene (Scheme 1) can enhance the biosynthesis of triterpenoids [64,65,66,69]. Along with overexpression, the enhancement can also be facilitated by inactivation of *TRP1* (phosphoribosylanthranilate isomerase), *GAL1* (galactokinase), and *GAL80* (galactose/lactose metabolism regulatory protein) involved in metabolic processes that “distract” cells from biosynthesis of triterpenoids [65,66]. Thus, overexpression of *tHMG1*, *ERG1*, and *ERG9* and inactivation of *GAL1* and *GAL80* in the chromosome of *Saccharomyces cerevisiae* JDY52 and its use in a 5-L fermenter with 40 g/L glucose resulted in 606.9 ± 9.1 mg/L OA after 144 h. To date, it is the highest yield reported [65]. In another study, the use of *Yarrowia lipolytica* ATCC 201249 with overexpressed *ERG1*, *ERG9*, *ERG20*, and *tHMG1* and the inserted expression modules *βAS* and *CYP716A2*-linker(GSTSSG)-*t46ATR1* (ATR1 with truncated *N*-terminal 46 amino acids) provided the yield of 540.7 mg/L OA after 82 h in a 5-L fermenter with 100 g/L glucose [69]. Despite the fact that *S*. *cerevisiae* JDY52 produced relatively higher OA amounts (606.9 ± 9.1 mg/L) [65], the productivity of *Y*. *lipolytica* was 6.59 mg/L/h and exceeded that of *S*. *cerevisiae* (4.214 mg/L/h).

Various techniques of yeast genome modification were also applied to intensify the biosynthesis of triterpenoids. It was shown that the expression of inserted plant genes from low-copy and single-copy plasmids was more effective than that from integrated, high-copy, and multicopy plasmids [71,73]. In Reference [71], the activities of *S*. *cerevisiae* TM30 and *S*. *cerevisiae* TM44 obtained from the same parent strain by including different plasmid variations were evaluated. In the first case, the strain expressing *CYP716Y1* and *CYP716A12* to obtain a self-processing polyprotein with two enzymes bound via oligopeptide 2A catalyzed the formation of β-amyrin (**6**), erythrodiol (**8**), OA, oleanolic aldehyde (**9**), and 16α-hydroxy-oleanolic aldehyde (**10**), while the second strain produced two self-processing polyproteins, one consisting of CYP716Y1 and CYP716A12 and the other consisting of AtATR1 and UDP-dependent glycosyl transferase UGT73C11, and catalyzed the formation of 3-*O*-Glc-echinocystic acid (**11**) and 3-*O*-Glc-OA (**12**).


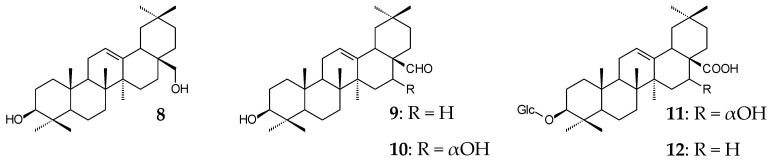


The most promising research area in this field is combinatorial biosynthesis. Change in the natural biosynthesis pathway or combination of synthesis genes from different sources leads to enhanced efficiency of microbial biosynthesis or to the formation of new compounds. The Moses group (2014) studied the C24-oxidizing activity of *CYP93E2* orthologs from different species of the family *Fabaceae*. The obtained strains catalyzed the formation of 24-hydroxy-β-amyrin (**13**). Almost all strains (except one containing *CYP93E7*) simultaneously catalyzed the formation of 3β-hydroxy-olean-12-en-24-oic acid (**14**). The highest conversion (79.4%) of β-amyrin (**6**) to 24-hydroxy-β-amyrin was observed using saccharomycetes containing *CYP93E9* (*P. vulgaris*) [72]. Dale et al. (2020) conducted a comparative study on catalytic activities of 12 βASs and 16 enzymes of the CYP716A subfamily. Of all the βASs studied, synthases derived from *Artemisia annua* and *Chenopodium quinoa* exhibited the highest catalytic activity (10.8 ± 1.0 mg/L β-amyrin). Comparatively, CYP716AL1 (*C. roseus*) showed the highest activity (14.3 ± 1.6 mg/L OA) of the 16 CYP716As studied. When a combination of genes encoding these enzymes was used, the OA yield made up 8.5 ± 0.2 mg/L. At the same time, saccharomycetes containing CYP716A75 (*Maesa lanceolata*), CYP716A79 (*C. quinoa*), CYP716A110 (*Aquilegia coerulea*), and CYP716A1 (*A. thaliana*) did not catalyze the formation of OA; erythrodiol (**8**) but not OA was detected in all cases [68]. Substitutions of *CYP88D6* (C11 oxidase) for *Unigene25647* (97% similarity) and *ATR1* (*A. thaliana*) for *GuCPR1* (*G. uralensis*) were performed, and a combination of *GuCPR1* and two *Unigene25647* cassettes was inserted into the genome of *S. cerevisiae* SGib. These manipulations resulted in the formation of 18.9 ± 2.0 mg/L GA and about 80.0 mg/L 11-oxo-β-amyrin (**15**) after 144 h of the fed-batch fermentation with ethanol (30 mL every 24 h) compared with the previously obtained 20.4 ± 7.7 µg/L GA and 0.5 ± 0.1 mg/L 11-oxo-β-amyrin (**15**) and β-amyrin (**6**) and trace amounts of 11α-hydroxy-β-amyrin (**16**), 30-hydroxy-11-oxo-β-amyrin (**17**), and glycyrrhetaldehyde (**18**) [62]. In strain *S. cerevisiae* BY-OA, substitution of *CYP716C49* (*C. pinnatifida*) for the homologue (47.9% similarity) *CaCYP716C49* (*C. asiatica*) allowed for obtaining 384.3 mg/L maslinic acid (**19**, 2α-hydroxy-OA) after 96-h incubation in a 5-L fermenter with glucose (5 g/L). When *S. cerevisiae* BY-T3 containing *mix-AS*, *VvCYP716A15*, *CPR*, and *CaCYP716C49* from different plant sources were used in the same conditions; corosolic acid (**20**, 2α-hydroxy-UA) was formed after 144 h [70]. The activity of various combinations of CYP450 from *M. truncatula* was studied in Reference [67]. The main products of synthesis in different cases were soyasapogenol B (**21**), gypsogenic acid (**22**), or 11-deoxo-GA (**23**). Importantly, saccharomycetes expressing *CYP716A12* and *CYP93E2* catalyzed the formation of 4-*epi*-hederagenin (**24**), and the yeasts expressing *CYP716A12* and *CYP72A63* catalyzed the formation of queretaroic acid (**25**). These compounds were not previously detected in *M. truncatula* tissues, indicating the great potential of combinatorial biosynthesis using microorganisms [67].


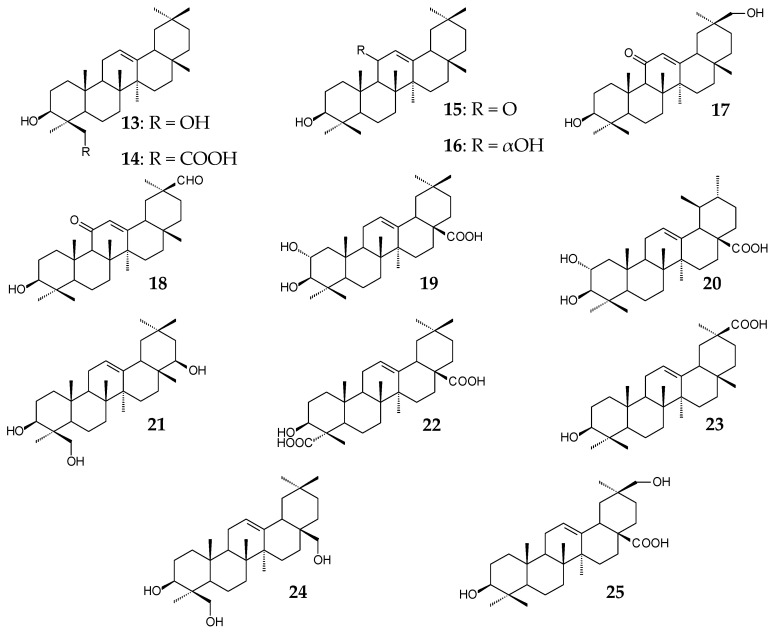


## 4. Biological Activities of Triterpenic Acids and Their Native Derivatives

Extracts obtained from plant sources using various solvents and containing the pentacyclic triterpenoids reviewed in this paper usually exhibit a wide range of biological properties. Methanolic OA-containing extracts from various parts of *Betula pendula* exhibited antibacterial activity against test cultures *Staphylococcus aureus* and *Bacillus subtilis* [3]. Methanolic extracts from the aerial part of the tropical plant *Baccharis uncinella* containing OA and UA showed antiparasitic activity by limiting the growth of promastigote and amastigote forms of *Leishmania amazonensis* and enhanced the immune response in infected mice [52]. The ethyl acetate fraction of *Glycyrrhiza uralensis* root extract inhibited TNF-α-induced activation of NF-κB in HepG2 cells [42]. The ethyl acetate fraction of *Potentilla fulgens* root extract containing ursane triterpenoids showed antioxidant activity by inhibiting the production of free radicals [58]. An alcohol extract and ethyl acetate fraction of *Fragaria ananassa* perianth containing various triterpenoids had a pronounced cytotoxic effect on B16-F10 melanoma cells and inhibited their melanogenesis by 79.1% and 80.2%, respectively [4]. The allelopathic effect (inhibition of seed maturation and root growth of nearby plants) of *Alstonia scholaris* was also due to high UA content (2.5 ± 0.6 mg/g dry weight of leaves) [49].

Isolation of every triterpenic acid individually allows studying their bioactive properties in detail and explaining the pharmacological properties of some plants. The antibacterial property of birch bark was determined by a high content of pentacyclic triterpenoids, in particular, OA, which exhibits a pronounced antibacterial activity against *S*. *aureus* (minimal inhibitory concentration (MIC) 1.25%) and *B*. *subtilis* (MIC 0.625%) [3]. Antimicrobial activity of GA was manifested as the ability to reduce the motility of *Pseudomonas aeruginosa* cells and the level of biofilm formation. This can make a significant contribution to the development of effective antibiotic-free therapy for *Pseudomonas* infections [74]. UA was able to inhibit both the growth of *Mycobacterium tuberculosis* in vitro [75] and the replication of rotavirus in a dose-dependent manner [76]. In addition, OA and UA were shown to inhibit the COVID-19 (SARS-CoV-2) main protease, a key enzyme of the virus replication, through *in silico* studies [77,78].

OA, as an antitumor agent, increased the sensitivity of sarcoma cells to chemotherapeutic drugs in human soft tissues [79]. UA had similar properties, significantly increased the effectiveness of colorectal cancer chemotherapy, and reduced its side effects in vitro and in vivo [80]. The cytotoxic effect of the extract of *Fragaria ananassa* perianth on B16-F10 melanoma cells was determined partly by the presence of cytotoxic UA that suppressed the melanin production by 40.2% [4]. Additionally, UA reduced the spread of human myeloma cells by inhibiting the deubiquitinating protease USP7 [81] and caused apoptosis of gastric cancer cells by activating the caspases poly (ADP-ribose) polymerase and by inducing the release of reactive oxygen species [6].

The OA hepatoprotective activity was shown to be related to its inhibitory effect against carboxylesterase (therapeutic target for hypertriglyceridemia) and the hepatitis C virus (HCV) [5,82]. GA exhibited the hepatoprotective effect by inhibiting NO formation in rat hepatocytes, *iNOS* suppression, and *COX-2* expression and by decreasing the activity of NF-κB transcription factor in HepG2 cells [42]. The ability of GA to stimulate a neuroprotective property of microglia and to suppress the MAPK signaling pathway of the central nervous system caused a decrease in the severity of experimental autoimmune encephalomyelitis in mice [83]. UA could be an effective antidiabetic agent due to its ability to inhibit α-glucosidase activity [60]. OA and UA exhibited their inhibitory effects against lypopolysaccharide (LPS)-induced NO production in RAW 264.7 cells that determined their anti-inflammatory activity [7].

Along with their parent acids, the native derivatives also exhibit pronounced biological activities. Thus, the OA derivatives 22β-acetoxy-3,25-epoxy-3α-hydroxyolean-12-en-28-oic acid (**26**) and methyl 3,25-epoxy-3α-hydroxy-11-oxo-22β-senecioyloxyolean-12-en-28-oate (**27**) isolated from *Lantana camara* herb extract demonstrated antibacterial activity against a number of gram-positive and gram-negative bacteria [84].


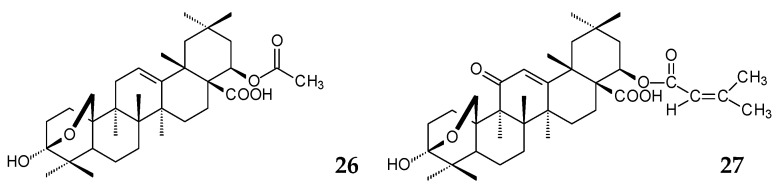


Oleanane 27-carboxy derivatives (**28**‒**30**) isolated from *Chrysosplenium carnosum* exhibited pronounced inhibitory activities against the mouse melanoma cell lines B16F10 and SP2/0 [85]. Natural 2α-hydroxylated OA derivative (**20**, corosolic acid) had cytotoxic effects on CaSki cells (human cervical cancer) by inducing apoptosis, by arresting the cell cycle in the G2/M phase, and by inhibiting the PI3K/Akt signaling pathway [86]. 2,3-seco-Derivative of the ursane type (**31**) from *Siphonodon celastrineus* showed pronounced cytotoxic activity against MOLT-3 cancer cells [87].


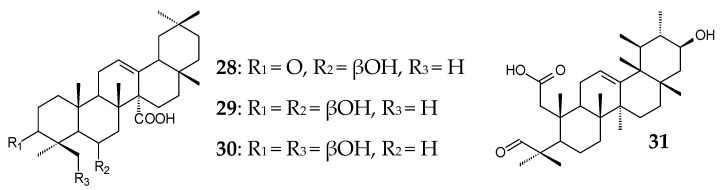


Oleanane derivatives from *Panax stipuleanatus* (C3, C28-diglycosides **32**–**34**) and *Astilbe rivularis* (3β-*trans*-*p*-coumaroyloxy-olean-12-en-27-oic acid (**35**) and 6β-hydroxy-3-oxoolean-12-en-27-oic acid (**36**)) as well as oleanane and ursane polyhydroxylated derivatives (**37**–**40**) from *Rosa laevigata* exhibited anti-inflammatory activities [88,89,90] due to the suppression of TGFBIp-mediated hyperpermeability in vitro and in vivo as well [90]. Ursane derivatives from *Durio zibethinus* were shown to exhibit a more pronounced anti-inflammatory effect in LPS-induced NO production in RAW 264.7 cell inhibition tests compared to oleanane triterpenoids, for which the activity was reduced by C2 hydroxylation [7].


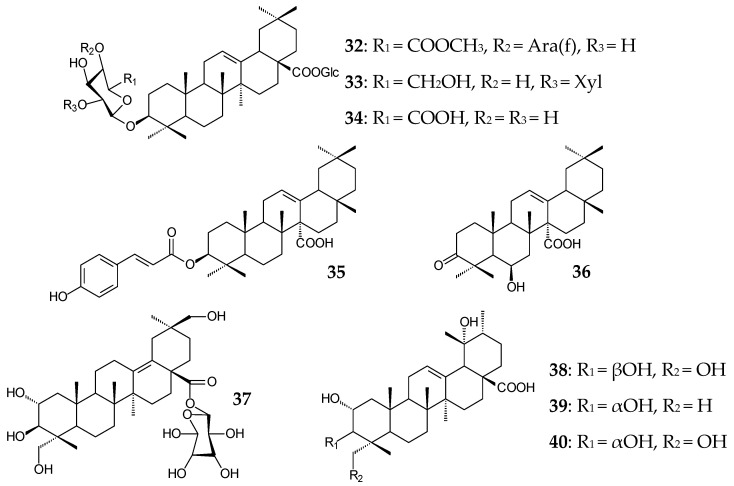


The pronounced inhibitory activity of *Cecropia telentida* root extracts against 11β-hydroxysteroid dehydrogenase could be determined by the presence of a new, probably 2α,20β-dihydroxylated UA derivative (**41**, isoyarumic acid) [91]. Ursane type 2,3-dihydroxy derivatives fulgic acids A (**42**) and B (**43**) isolated from *Potentilla fulgens* exhibited antioxidant effect by inhibiting the formation of free radicals [58]. Antiviral, antibacterial, hepatoprotective, anti-inflammatory, and antitumor activities of root extracts of *Bupleurum chinense* and *B*. *scorzonerifolium* species that are widespread in China were partially provided by the presence of bioactive oleanane saikosaponins (**44**–**46**) [92].


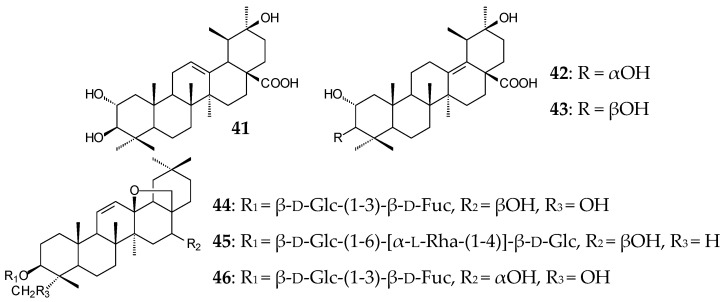


Due to the wide distribution and availability of the pentacyclic triterpenoids discussed in the review, they are often used to synthesize new semi-synthetic derivatives with antibacterial [93], antiviral [10,82,94], anti-inflammatory [95,96], hepatoprotective [5], antitumor [8,97,98,99], and other activities. Biological activities of oleanane and ursane semi-synthetic derivatives were studied in a number of reviews summarizing the data on derivative types [100,101,102] and biological activities [103,104,105].

## 5. Biological Transformation

Taking into account the relative availability of the discussed triterpenic acids in natural sources and their high bioactivity, it is interesting to assess the possibility of directed transformations of these compounds to expand the range of biologically active compounds and to increase their bioavailability. Chemical methods are currently the most tested and used to transform acids **1**–**3**. However, chemical methods often require extreme acidity and temperature values, expensive catalysts, or protective groups of molecule reactive centers [8,9,10,106]. In contrast, biological transformation processes do not use aggressive reagents and can occur under normal eco-friendly conditions. Moreover, microorganisms are able to catalyze a wide range of regio- and stereoselective reactions that are difficult to perform chemically [12].

One of the most promising ways to highlight the pharmacological potential of native pentacyclic triterpenoids is the functionalization of their molecules by polyhydroxylation. Such functionalized derivatives hydroxylated by plant P450-dependent monooxygenases [107] are widespread in nature but are usually found in trace amounts or as part of a difficult-to-separate mixture. Enzymatic activity of microorganisms used for transformation of pentacyclic triterpenoids allows for obtaining hydroxylated derivatives with high yield and regioselectivity. Moreover, microbial hydroxylation occurs not only in the A ring but also at hard-to-reach positions on the B, D, and E rings. In addition to hydroxylation, microbial functionalization of pentacyclic triterpenoids can occur by less frequent reactions of carboxylation, glycosylation, lactone formation, and others.

### 5.1. Fungal Transformation

The described biotransformation processes of the compounds discussed in this review often occur using mycelial fungi of various species from the phyla *Ascomycota* (orders *Glomerellales*, *Hypocreales*) and *Mucoromycota* (order *Mucorales*). Fungal conversions of these compounds are accompanied by the formation of derivatives with hydroxyl groups at C1, C7, C15, C21, C24, or C30; oxo groups at C3, C7, or C21; glucopyranoside groups at C3, C28, or C30; lactone groups at C28/C13 or C3/C4, etc. as well as by the A ring fragmentation. The acid concentration used in biotransformation experiments usually ranges from 0.02 g/L to 1.0 g/L. The yield of transformation products (1.0% to 77.5%) and the duration of the processes (2 to 20 days) vary depending on the fungal catalyst characteristics (Table 3).

Abundantly found in nature, *Rhizomucor miehei* CECT 2749 partially metabolized OA (approximately 0.5 g/L) for 13 days to form of 1β,30-dihydroxy-OA (**47**), 7β,30-dihydroxy-OA (**48**), and 30-hydroxy-OA (**25**) in equivalent amounts (5.0‒6.0%) [108]. Compound **25**, known as queretaroic acid, was first isolated from *Lemaireocereus queretaroensis* and *L*. *beneckei* endemic to Mexico [109]. This compound was shown to exhibit a moderate antitumor activity against HeLa cells [110]. Queretaroic acid **25** (3.3%) was obtained by the 24-h transformation of OA (0.2 g/L) by *Escherichia coli* cells expressing *Nonomuraea recticatena* CYP450 *moxA* and *Pseudomonas* redox partner *camAB*. At the same time, the use of cell-free reaction systems allowed to increase the yield of compound **25** to 17.0% [111]. Transformation of OA methyl ester (approximately 0.3 g/L) by *R*. *miehei* CECT 2749 during 13 days was accompanied by 7,30-dihydroxylation and, in addition, a 9(11),12-diene moiety formation in the C ring, producing 15.0% methyl 3β,7β,30-trihydroxy-oleane-9(11),12-dien-28-oate (**49**) [112].


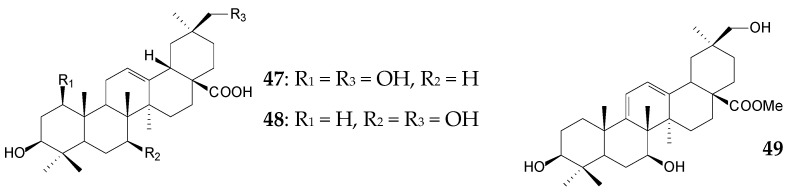


*Colletotrichum lini* AS 3.4486 was shown to catalyze the C15 hydroxylation of OA to form 15α-hydroxy-OA (**50**) [113]. In turn, ascomycete *Trichothecium roseum* (M 95.56) [114] and mucoromycete *Circinella muscae* AS 3.2695 [15] catalyzed the oxidation of OA (0.08 g/L and 0.02 g/L, respectively) to form 7β,15α-dihydroxy-3-oxo-olean-12-en-28-oic acid (**51**) on day 6 (7.5%) and day 7 (6.1%), respectively. At the same time, an intermediate (6.25%) of the dihydroxylation process—15α-hydroxy-3-oxo-olean-12-en-28-oic acid (**52**)—was also isolated from the culture medium of *T. roseum* (M 95.56) [114]. *C*. *muscae* AS 3.2695 simultaneously catalyzed a wide variety of hydroxylation and glycosylation reactions with the formation of 7β-hydroxy-OA (**53**), 7β,21β-dihydroxy-OA (**54**), 7α,21β-dihydroxy-OA (**55**), 7β,15α-dihydroxy-OA (**56**), 7β-hydroxy-3-oxo-olean-12-en-28-oic acid (**57**), 7β,15α-dihydroxy-OA 28-*O*-β-d-glucopyranosyl ester (**58**), 21β-hydroxy-OA 28-*O*-β-d-glucopyranosyl ester (**59**), and OA 28-*O*-β-d-glucopyranosyl ester (**60**) ranging from 3.1% to 5.8% [15]. C7 hydroxylation and C28 glycosylation presumably contributed to an increase in the anti-inflammatory activity of derivatives, while C21 hydroxylation led to a decreased ability of compounds to inhibit the release of LPS-induced nitric oxide by RAW 264.7 cells [15].


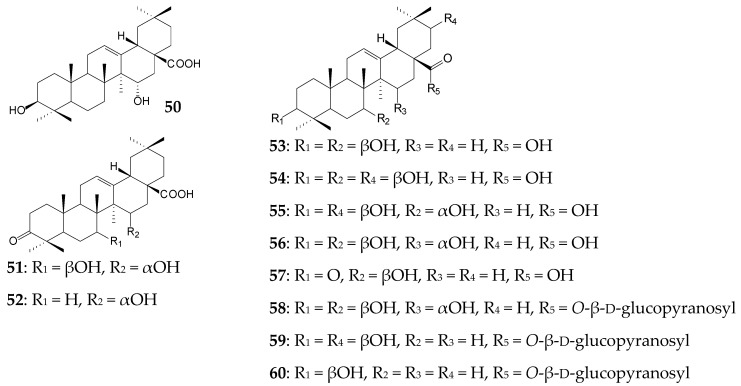


*C*. *muscae* AS 3.2695 was also active against GA (approximately 0.06 g/L) and initiated oxidation, acetylation, and glycosylation reactions with the formation of metabolites (yield did not exceed 2.4%) on day 7. The metabolites included 7β-hydroxy-GA (**61**), 15α-hydroxy-GA (**62**), 7β,15α-dihydroxy-GA (**63**), 3,11-dioxo-7β-hydroxy-18β-olean-12-en-30-oic acid (**64**), 7β,15α-dihydroxy-3,11-dioxo-18β-olean-12-en-30-oic acid (**65**), 7β-hydroxy-11-oxo-18β -olean-12-en-30-oic acid 3-*O*-β-d-glucopyranoside (**66**), 7β-hydroxy-11-oxo-18β-olean-12-en-30-oic acid 3-*O*-β-d-6′-*O*-acetyl-glucopyranoside (**67**), 15α-hydroxy-11-oxo-18β-olean-12-en-30-oic acid 3-*O*-β-d-glucopyranoside (**68**), 15α-hydroxy-11-oxo-18β-olean-12-en-30-oic acid 3-*O*-β-d-6′-*O*-acetyl-glucopyranoside (**69**), and 7β-hydroxy-GA 30-*O*-β-D-glucopyranoside (**70**) [115]. The above GA derivatives inhibited LPS-induced NO release by RAW 264.7 cells to different extents. Moreover, compounds **61** and **64** were shown to exhibit antimicrobial activity against the antibiotic-resistant strain *Enterococcus faecalis* [116], while compound **63** exhibited antioxidant and hepatoprotective properties [117].


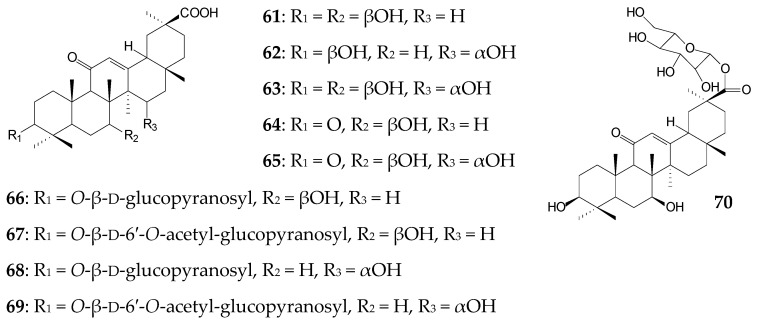


The same authors reported *Rhizopus arrhizus* AS 3.2893 to perform oxidative transformation of GA (approximately 0.06 g/L) at C3, C7, and C15 for 7 days with the formation of 7β-hydroxy-GA (**61**), 15α-hydroxy-GA (**62**), 7β,15α-dihydroxy-GA (**63**), 3β-acetoxy-7β-hydroxy-11-oxo-18β -olean-12-en-30-oic acid (**71**), 7-oxo-GA (**72**), 7α-hydroxy-GA (**73**), and 15α-hydroxy-7-oxo-GA (**74**) (the yield of each compound did not exceed 2.8%). They also exhibited anti-inflammatory effects in the LPS-induced NO production inhibition test in RAW 264.7 cells [115].


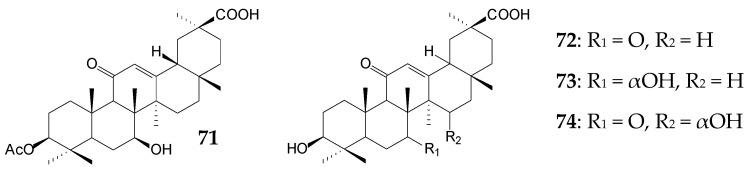


Biological hydroxylation of GA at concentrations of 0.25 g/L (48 h) and 1.0 g/L (14 days) at C7 and C15 producing the main metabolite 7β,15α-dihydroxy-GA (**63**) was also catalyzed by *C*. *lini* AS 3.4486 [118] and *Absidia pseudocylinderospora* ATCC 24169 [117], respectively. Moreover, *A. pseudocylinderospora* ATCC 24169, during long-term cultivation (14 days), catalyzed the formation of more than 18.0% of this compound (**63**) possessing antioxidant, hepatoprotective [117], and anti-inflammatory [115] activities.

Preparative biotransformation of 0.1 g/L GA by *Cunninghamella blakesleana* CGMCC 3.970 for 7 days resulted in a mixture of 15α,24-dihydroxy-GA (**75**), 15α,24-dihydroxy-3,11-dioxo-18β -olean-12-en-30-oic acid (**76**), 7β,24-dihydroxy-3,11-dioxo-18β-olean-12-en-30-oic acid (**77**), 3,11-dioxo-7β,15α,24-trihydroxy-18β-olean-12-en-30-oic acid (**78**), and 7α,24-dihydroxy-3,11 -dioxo-18β-olean-12-en-30-oic acid (**79**). The yield of each acid did not exceed 1.3% [14]. Compounds **75**, **78**, and **79** were found to effectively inhibit LPS-induced NO production in mouse microglia cells with IC_50_ values of 0.76 mmol/L, 0.94 mmol/L, and 0.16 mmol/L, respectively [14]. Interestingly, when *C*. *blakesleana* AS 3.970 was used, an increase in GA concentration to 0.3 g/L led to accumulation of two main products 7β-hydroxy-GA (**61**, 30.0%) and 3,11-dioxo-7β-hydroxy-18β-olean-12-en-30-oic acid (**64**, 25.0%) with pronounced antibacterial activity after 5 days [116]. In the case of *C*. *elegans* TSY-0865, only 2.5% of 7β-hydroxy-GA (**61**) was formed during 8 days [119].


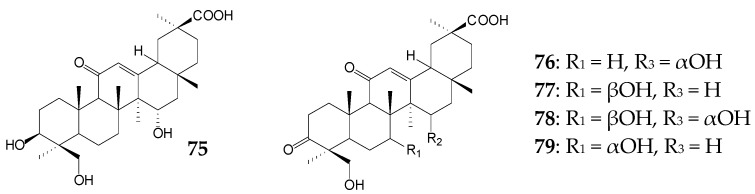


UA biotransformation processes include oxidation and lactone formation reactions. Thus, *Syncephalastrum racemosum* CGMCC 3.2500 transformed approximately 0.1 g/L UA to 7β,21β-dihydroxy-UA (**80**, 12.9%), 1β,21β-dihydroxy-UA (**81**, 3.9%), 1β-hydroxy-21-oxo-UA (**82**, 12.1%), 3β,21β-dihydroxy-urs-11-en-28,13-olide (**83**, 3.4%), and 3β,7β,21β-trihydroxy-urs-11-en-28,13-olide (**84**, 2.9%) within 10 days [13]. Similar bioconversion processes have been previously demonstrated using another strain *S*. *racemosum* AS 3.264 [120]. UA derivatives with a rare lactone moiety were shown to exhibit moderate inhibitory activity against HCV [13].


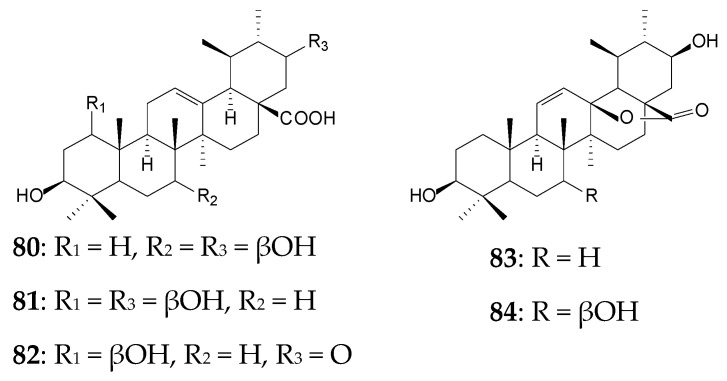


For 20 days, *Gliocladium roseum* CGMCC 3.3657 catalyzed oxidative transformation of the A ring of UA (0.1 g/L) by Baeyer–Villiger-type reaction and C21 oxidation to form 21β-hydroxy-3-oxo-urs-12-en-3,4-olide-28-oic acid (**85**, 8.0%), 3,21-dioxo-urs-12-en-3,4-olide-28-oic acid (**86**, 6.25%), 21β-hydroxy-3,4-seco-ursane-4(23),12-dien-3,28-dioic acid (**87**, 1.5%), and 21-oxo-3,4-seco-ursane-4(23),12-dien-3,28-dioic acid (**88**, 1.0%). Derivatives **86** and **88** containing a 21-oxo group showed the most pronounced anti-HCV activity [121].


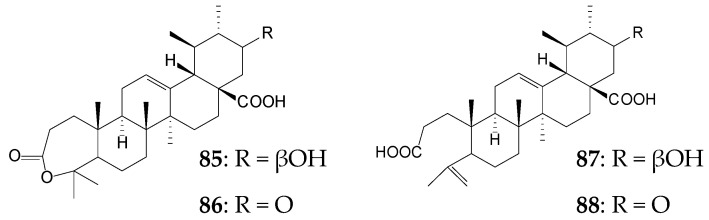


When *Mucor spinosus* AS 3.3450 was used to bioconvert UA, three metabolites, 7β-hydroxy-UA 28-ethanone (**89**, 5.04%), 7β,21β-dihydroxy-UA (**81**, 1.64%), and 21β-hydroxy-urs-12-en-28-oic acid 3-*O*-β-D-glucopyranoside (**90**, 2.13%), were formed within 96 h. Compound **89** was shown to exhibit pronounced (higher than that of UA) cytotoxic activity against HeLa, K562, and KB tumor cell lines [122].


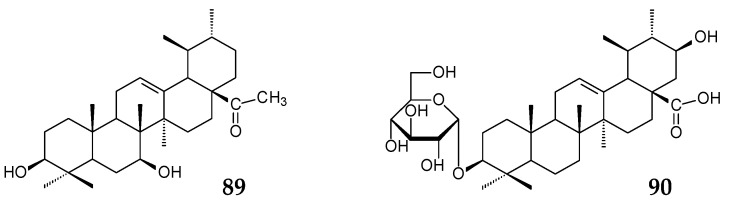


### 5.2. Bacterial Transformation

The literature describes a few cases of pentacyclic triterpenoid bioconversion using gram-positive bacteria of the genera *Bacillus*, *Nocardia*, and *Streptomyces* and accompanied by the formation of C1, C2, C7, C11, C21, C24, or C29 hydroxylated derivatives, derivatives with a methyl ester group at C28; oxogroup at C3; additional carboxyl groups at C29 or C30; glucopyranoside groups at C3, C28, or C30; lactone group at C28/C13; and derivatives with a fragmented A ring. In biotransformation experiments, the compounds are usually used in concentrations ranging from 0.04 g/L to 0.3 g/L, and the yield of derivatives ranges from 5.0% to 60.0%. The duration of bioconversion is 3 to 5 days; only in the case of using *Nocardia*, it reached 13 days (Table 3).

Actinobaceria of the genus *Nocardia* were capable of selective methylation of the C28-carboxylic group of pentacyclic triterpenoids [123]. The use of resting or immobilized cells of *N*. *iowensis* DSM 45197 as biocatalysts of the OA (approximately 0.3 g/L) transformation process for 13 days resulted in the formation of methyl OA (**91**) as the main bioconversion product (more than 60.0%), small amounts (≤5.0%) of methyl 3-oxo-olean-12-en-28-oat (**92**), and metabolite **93** unidentified by the authors [16]. 3-oxo-OA (**92**) was shown to have pronounced antimelanoma [124], antileishmanial, and antitrypanosomal effects [125]. Despite numerous successful examples to increase the efficiency of the biotransformation process by immobilizing microbial cells [126], the use of fixed *Nocardia* cells in alginate carriers led to a decrease in their catalytic activity, as confirmed by a 10-fold decrease in the formation of compound **91** and only a short-term occurrence of compound **93** in the culture medium [16]. The ability of *Nocardia* sp. to transform UA by methylation, by C3 oxidation, and by formation of the enone moiety in the A ring was previously shown. It was noted that the biotransformation process did not depend on the composition of the culture medium used, while the temperature increase (from 28 °C to 36 °C) for actinobacteria cultivation contributed to a 2-fold increase in the reaction rate [127].


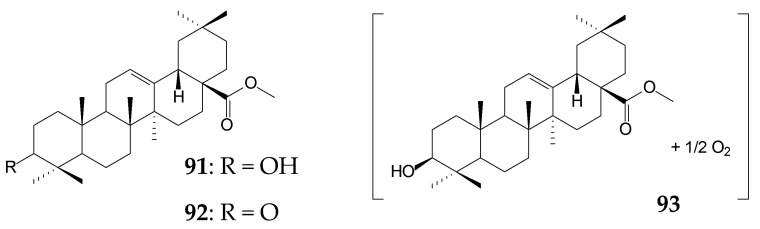


The bacterial culture of *Streptomyces griseus* ATCC 13273 catalyzed hydroxylation and site-selective oxidation of the C29 methyl group of OA (0.04 g/L) to the carboxyl group within 5 days to form 3β-hydroxy-olean-12-ene-28,29-dioic acid (**94**, 21.9%), 3β,24-dihydroxy-olean-12-ene-28,29-dioic acid (**95**, 32.7%), and 3β,21β,24-trihydroxy-olean-12-ene-28,29-dioic acid (**96**, 5.9%). Hydroxylation at C21 was shown to increase the anti-inflammatory activity of OA derivatives [128]. Using the same strain, biotransformation of OA (approximately 0.05 g/L) with the formation of derivatives **94** and **96** was previously described by Y. Zhu et al. [129].


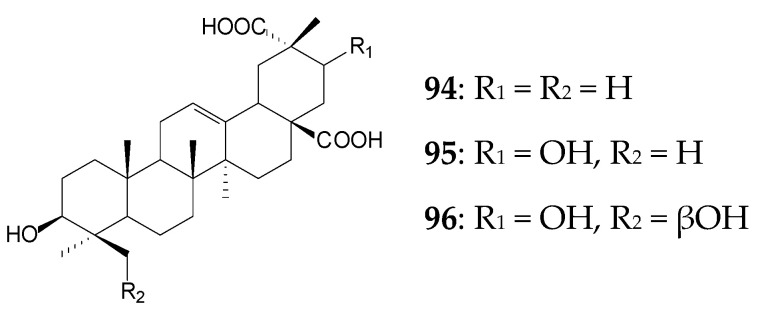


In addition to OA, *S*. *griseus* ATCC 13273 cells can also transform UA (0.04 g/L) by catalyzing site-selective oxidation of the C30 methyl group to the carboxyl one and C24-hydroxylation within 3 days to produce 3β-hydroxy-urs-12-ene-28,30-dioic acid (**97**) and 3β,24-dihydroxy-urs-12-ene-28,30-dioic acid (**98**), with the product yield exceeding 30.0%. Transformation of UA derivatives 3-oxo-UK (**99**) and 2α-hydroxy-UA (**20**, corosolic acid) at a concentration of 0.04 g/L by this actinobacterial strain also occurred by selective C30-oxidation and hydroxylation, that led to a mixture of 3-oxo-urs-12-ene-28,30-dioic acid (**100**, 24.1%) and 24-hydroxy-3-oxo-urs-12-ene-28,30-dioic acid (**101**, 45.9%) in the former case and a mixture of 2α,3β-dihydroxy-urs-12-ene-28,30-dioic acid (**102**, 29.0%) and 2α,3β,24-trihydroxy-urs-12-ene-28,30-dioic acid (**103**, 15.9%) in the latter case [17].


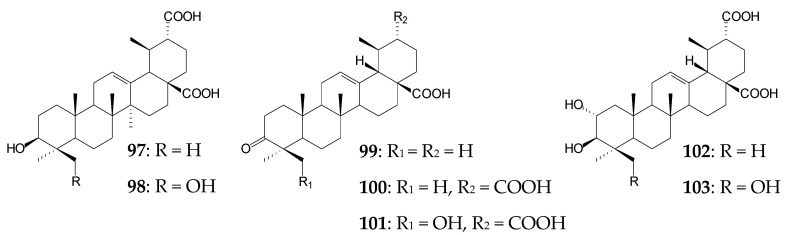


A gram-positive *Bacillus megaterium* CGMCC 1.1741 was able to transform UA (0.2 g/L), generating the main derivative 1β,11α-dihydroxy-UA (**104**, 26.87%) and minor derivatives (5.03–13.50%) 3-oxo-urs-12-en-28-oic acid (**99**), 1β,11α-dihydroxy-3-oxo-urs-12-en-28-oic acid (**105**), 1β-hydroxy-3-oxo-urs-12-en-28,13-olide (**106**), and 1β,11α-dihydroxy-3-oxo-urs-12-en -28-*O*-β-D-glucopyranoside (**107**) over 4 days. Derivatives **105** and **106** were shown to effectively inhibit LPS-induced NO release in RAW 264.7 cells (IC_50_ 1.71 µmol and 1.24 µmol, respectively) [18], and derivative **99** was shown to inhibit cathepsin L-like rCPB2.8 protease of *Leishmania mexicana* [130].


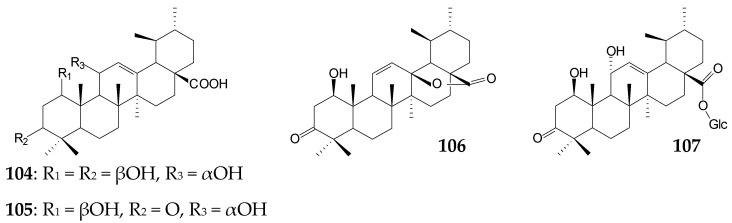


Xu et al. (2020) have recently used the tandem biotransformation of oleanane-type pentacyclic triterpenoids using the fungal strain *Rhizopus chinensis* CICC 40335 and bacterial strains *Bacillus subtilis* ATCC 6633 and *Streptomyces griseus* ATCC 13273 [34]. The primary transformation of OA (0.2 g/L) using *R*. *chinensis* CICC 40335 occurred within 4 days by the formation of 7β,21β-dihydroxy-OA (**54**, 53.75%) previously obtained using the fungal strains *Mucor rouxii* NRRL 1894 [131] and *Circinella muscae* AS 3.2695 [15]. Further 4-day biotransformation of the obtained compound (**54**) led to the formation of 7β,21β-dihydroxy-olean-12-en-28-oic acid 3-*O*-β-D-glucopyranoside (**108**, 46.5%) using *B*. *subtilis* ATCC 6633 cells and a mixture of 7β,21β,29-trihydroxy-OA (**109**, 26.0%) and 3β,7β,21β-trihydroxy-olean-12-ene-28,29-dioic acid (**110**, 15.0%) using *S*. *griseus* ATCC 13273 cells [34].

Bioconversion of GA (0.2 g/L) using *R*. *chinensis* CICC 40335 occurred by selective oxidation with the formation of 7β-hydroxy-GA (**61**, 77.5%) on day 4 [34]. Note that the C7-hydroxylation process is typical for many cultures, for example, *C*. *muscae* AS 3.2695, *Rhizopus arrhizus* AS 3.2893 [115], and representatives of the genus *Cunninghamella* [116,119]. Further addition of GA or compound **61** in the culture medium of *B*. *subtilis* ATCC 6633 led to the formation of 30-*O*-β-d-glucopyranoside derivatives (**111** (27.5%) and **68** (44.0%), respectively) previously obtained by B. Fan et al. [115]. Assessment of the neuroprotective potential of the obtained OA and GA derivatives revealed that glycosylation significantly contributed to a decrease in the neuroprotective activity of compounds while carboxylation led to a significant increase in the neuroprotective effect of OA derivatives [34].


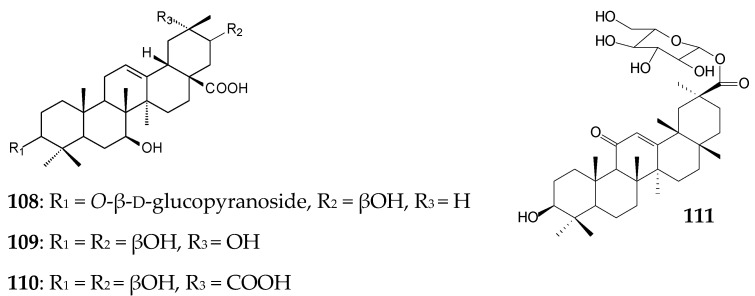


## 6. Conclusions

Triterpenoids are secondary metabolites of plants, fungi, marine invertebrates, and algae that are formed during cyclization of an acyclic triterpene squalene [132,133,134,135,136,137,138]. According to the number of cycles, triterpenes and triterpenoids are divided into several groups; the most numerous are pentacyclic triterpenic derivatives [139]. In nature, this group is most widely represented by compounds of oleanane (OA and GA) and ursane (UA) types, which in large quantities can accumulate in various parts of higher plants [39,41,46]. In addition, the biosynthesis of these compounds can be carried out in microbial cells able to catalyze the MVA pathway and to be genetically modified using plant genes [64,68,69,70]. The main difficulties of microbial biosynthesis are generally considered to be complexity and long duration of processes of searching for terpenoid synthesis genes of plants, their isolation, and the preparation of genetically modified microorganisms. The rapid development of bioinformatics methods, sequencing techniques, and *de novo* DNA synthesis significantly simplified the abovementioned processes and gave a new impetus to research in this field [140]. With the close cooperation of biochemists, microbiologists, and genetic scientists, microbial biosynthesis can become a promising technology for obtaining valuable pentacyclic triterpenoids. The compounds discussed in the review exhibit antitumor, antiviral, hepatoprotective, neuroprotective, and other activities [5,7,42,60,74,82,83]. Despite the wide range of known biological properties, the use of pentacyclic triterpenoids in pharmacology and medicine is limited because of their high hydrophobicity. The solution to this problem might be the synthesis of triterpenic derivatives with increased bioactivity, solubility, and bioavailability [5,82,93,94,141].

Studies of the possibility of obtaining new OA, GA, and UA derivatives by directed biotransformations should be considered a promising area. Over 20 examples of biotransformations of these compounds using fungal and bacterial cultures most often catalyzing hydroxylation have been described since 2013. Less frequently, the literature describes processes of deeper oxidation of triterpenoids as well as their glycosylation, esterification, acetylation, or carboxylation. Biocatalytic formation of triterpenic lactones or their derivatives with fragmented C–C bond was reported only in a few cases using UA [13,121]. In the biotransformation processes employing fungi, the degree of triterpenic acid conversion usually ranges from 2.6% to 77.5%, with an initial concentration of 0.02 g/L to 1.0 g/L, whereas in bacterial transformations, the degree of conversion reaches 27.5‒70.0%, with an initial concentration of 0.04‒0.3 g/L. When analyzed, the data showed that the biotransformation of oleanane and ursane pentacyclic triterpenoids led to derivatives with antioxidant, anti-inflammatory, antiviral, antitumor, antiparasitic, antimicrobial, neuroprotective, and hepatoprotective properties (Table 3). Provided more active development as an interdisciplinary tool, this method of obtaining biologically active compounds and their intermediates seems to be a promising strategy to design new medicinal agents against cancer and neurodegenerative diseases as well as potent antibacterial drugs against antibiotic-resistant pathogenic strains of microorganisms. By combining methods of microbial synthesis of native pentacyclic triterpenoids and their subsequent microbial transformations into bioavailable compounds, the industrial microbiology could provide a cycle of production of valuable biologically active substances. However, it should be noted that the described microbial catalysts have significant drawbacks. Fungi usually demonstrate mycelial growth type and form spores and mycotoxins, whereas few bacterial catalysts described are mainly represented by species, with their individual strains being pathogens. In this context, it is essential to conduct further in-depth studies of the processes of biological transformation of pentacyclic triterpenoids and to search for new nonpathogenic bacterial strains able to carry out highly effective synthesis of triterpenic derivatives with pronounced biological activities.

## Figures and Tables

**Table 1:** Some plant sources of oleanolic acid (OA), ursolic acid (UA), and glycyrrhetinic acid (GA).

Compound	Plant Source	Part of Plant	Reference
*Apocynaceae* Juss., nom. cons.			
OA, UA	*Alstonia scholaris* (L.) R.Br.	Leaves	[49]
OA, UA	*Plumeria obtusa* L. var. *sericifolia*	Leaves	[50]
*Aquifoliaceae* DC. ex. A.Rich.			
OA, UA	*Ilex guayusa* Loes.	Leaves	[39]
*Araliaceae* Juss., nom. cons.			
OA	*Panax stipuleanatus* H.T.Tsai & K.M.Feng	Roots	[51]
*Asteraceae* Bercht. & J.Presl, nom. cons.			
OA	*Baccharis uncinella* DC.	Leaves	[52]
*Betulaceae* Gray			
OA	*Betula pendula* Roth.	Bark	[3,53]
*Cornaceae* Bercht. & J.Presl, nom. cons.			
UA	*Cornus officinalis* Torr. ex Dur.	Seeds	[54]
*Ebenaceae* Gürke, nom. cons.			
OA, UA	*Diospyros* L.	Fruits	[55]
*Ericaceae* Juss., nom. cons.			
OA, UA	*Arctostaphylos uva-ursi* (L.) Spreng.	Fruits	[39]
OA, UA	*Vaccinium vitis-idaea* L.	Fruits	[53]
*Fabaceae* Lindl.			
GA	*Glycyrrhiza glabra* L.	Roots	[41]
	*Glycyrrhiza uralensis* Fisch.	Roots	[42]
	*Glycyrrhiza inflate* Batalin	Roots	[43]
*Gentianaceae* Juss., nom. cons.			
OA, UA	*Comastoma pulmonaria* Turcz.	Sprouts, roots	[38]
	*Swertia racemosa* Wall. ex Griseb.	Sprouts, roots	
*Lamiaceae* Martinov			
OA, UA	*Ocimum tenuiflorum* L.	Leaves	[39]
	*Lavandula angustifolia* Mill.	Flowers	
	*Origanum vulgare* L.	Leaves	
	*Rosmarinus officinalis* L.	Leaves	
	*Salvia officinalis* L.	Leaves	
	*Dracocephalum tanguticum* Maxim	Sprouts, roots	[38]
*Lythraceae* J.St.-Hil.			
OA, UA	*Punica granatum* L.	Fruits	[38]
	*Punica granatum* L.cv. Daqingpi	Flowers	[56]
*Malvaceae* Juss., nom. cons.			
OA	*Durio zibethinus* Murr.	Fruits	[7]
*Myrtaceae* Juss., nom. cons.			
OA, UA	*Syzygium aromaticum* (L.) Merr. & L.M.Perry	Flowers	[39]
	*Eucalyptus globules* Labill.	Leaves	
*Oleaceae* Hoffmanns. & Link, nom. cons.			
OA	*Olea europaea* L.cv. Picual	Fruits, leaves	[40]
	*Olea europaea* L. cv. Cornezuelo		
OA, UA	*Olea europaea* L.	Leaves	[39]
*Papaveraceae* Juss.			
OA, UA	*Meconopsis henrici* Bureau & Franch.	Sprouts, roots	[38]
	*Corydalis impatiens* (Pall.) Fisch. ex DC.	Sprouts, roots	
*Pinaceae* Lindl.			
UA	*Picea abies* (L.) H.Karst.	Resin, cons, sprouts	[57]
*Rosaceae* Juss.			
OA, UA	*Malus* P. Mill.	Fruits	[55]
	*Pyrus* L.	Fruits	
UA	*Fragaria ananassa* (Duchesne ex Weston) Duchesne ex Rozier	Perianth	[4]
UA	*Potentilla fulgens* Wall. ex Hook.	Roots	[58]
OA, UA	*Eriobotrya japonica* (Thunb.) Lindl	Leaves	[59]
*Rubiaceae* Juss., nom. cons.			
OA	*Uncaria laevigata* Wall. ex G.Don	Stem bark	[60]
UA	*Emmenopterys henryi* Oliv.	Leaves, sprouts	[61]
*Vitaceae* Juss., nom. cons.			
OA	*Vitis vinifera* L.	Fruits	[55]

Note: GA, glycyrrhetinic acid; OA, oleanolic acid; UA, ursolic acid.

**Table 2:** Biosynthesis of pentacyclic triterpenoids using engineered yeasts.

Parent Strain	Modification	Product (Yield)	Reference
*Saccharomyces cerevisiae* INVSc1	Insertion of *βAS*, *CPR* (*Lotus japonicus*), *CYP93E2*, and *CYP72A61* (*Medicago truncatula)*	β-Amyrin (**6** **, 1.07 mg/L), 24-hydroxy-β-amyrin (**13**, 0.27 mg/L), and soyasapogenol B (**21**, 1.35 mg/L)	[67]
	Insertion of *βAS*, *CPR* (*L*. *japonicus*), *CYP716A12*, and *CYP72A68* (*M*. *truncatula*)	β-Amyrin (**6**, 0.55 mg/L), erythrodiol (**8**, 0.09 mg/L), and gypsogenic acid (**22**, 0.96 mg/L)	
	Insertion of *βAS*, *CPR* (*L*. *japonicus*), *CYP716A12*, and *CYP93E2* (*M*. *truncatula*)	Erythrodiol (**8**), OA, 4-*epi*-hederagenin (**24**), and others (trace amount)	
	Insertion of *βAS*, *CPR* (*L*. *japonicus*), *CYP72A63*, and *CYP716A12* (*M*. *truncatula*)	Erythrodiol (**8**), OA, queretaroic acid (**25**), and others (trace amount)	
	Insertion of *βAS*, *CPR* (*L*. *japonicus*), *CYP93E2*, and *CYP72A63* (*M*. *truncatula*)	Probable 11-deoxo-GA (**23**) and others (trace amount)	
*S*. *cerevisiae* BY4742	Insertion of *βAS* (*Glycyrrhiza glabra*)	β-Amyrin (**6**, 36.2 ± 3.9 mg/L)	[71]
	Insertion of *βAS* (*M*. *truncatula*)	β-Amyrin (**6**, 19.0 ± 1.0 mg/L)	
*S*. *cerevisiae* TM3	Insertion of *AtATR1* (*Arabidopsis thaliana*), *CYP716Y1* (*Bupleurum falcatum*), and *CYP716A12* (*M*. *truncatula*)	β-Amyrin (**6**), erythrodiol (**8**), OA (1), oleanolic aldehyde (**9**), and 16α-hydroxy-oleanolic aldehyde (**10**)	
	Insertion of *CYP716Y1* (*B*. *falcatum*), *CYP716A12* (*M*. *truncatula*), *AtATR1* (*A*. *thaliana*), and *UGT73C11* (*Barbarea vulgaris*)	3-*O*-Glc-Echinocystic acid (**11**), 3-*O*-Glc-OA (**12**)	
	Insertion of *AtATR1* (*A*. *thaliana*) and *CYP93E2* (*M*. *truncatula*)	24-Hydroxy-β-amyrin (**13**, 1.3%)	[72]
	Insertion of *AtATR1* (*A*. *thaliana*) and *CYP93E7* (*Lens culinaris*)	24-Hydroxy-β-amyrin (**13**, 16.2%)	
	Insertion of *AtATR1* (*A*. *thaliana*) and *CYP93E8* (*Pisum sativum*)	24-Hydroxy-β-amyrin (**13**, 37.6%), probable 3β-hydroxy-olean-12-en-24-oic acid (**14**, 3.2%)	
	Insertion of *AtATR1* (*A*. *thaliana*) and *CYP93E5* (*Cicer arietinum*)	24-Hydroxy-β-amyrin (**13**, 51.3%), probable 3β-hydroxy-olean-12-en-24-oic acid (**14**, 7.6%)	
	Insertion of *AtATR1* (*A*. *thaliana*) and *CYP93E6* (*G*. *glabra*)	24-Hydroxy-β-amyrin (**13**, 50.2%), probable 3β-hydroxy-olean-12-en-24-oic acid (**14**, 6.3%)	
	Insertion of *AtATR1* (*A*. *thaliana*) and *CYP93E4* (*Arachis hypogaea*)	24-Hydroxy-β-amyrin (**13**, 47.0%), probable 3β-hydroxy-olean-12-en-24-oic acid (**14**, 5.5%)	
	Insertion of *AtATR1* (*A*. *thaliana*) and *CYP93E1* (*Glycine max*)	24-Hydroxy-β-amyrin (**13**, 58.8%), probable 3β-hydroxy-olean-12-en-24-oic acid (**14**, 13.5%)	
	Insertion of *AtATR1* (*A*. *thaliana*) and *CYP93E9* (*Phaseolus vulgaris*)	24-Hydroxy-β-amyrin (**13**, 67.6%), probable 3β-hydroxy-olean-12-en-24-oic acid (**14**, 11.8%)	
*S*. *cerevisiae* BY4742	Deletion of *TRP1*	Squalene (**4**, 9.6 mg/L)	[66]
*S*. *cerevisiae* BY4742-TRP	Overexpression of *tHMG1* and *LYS2*	Squalene (**4**, 150.9 mg/L)	
*S*. *cerevisiae* BY-T1	Insertion of *βAS* (*G*. *glabra*) Overexpression of *ERG1* and *ERG9*	β-Amyrin (**6**, 107.0 mg/L), squalene (**4**, 183.4 mg/L)	
	Insertion of *βAS* (*Panax ginseng*) Overexpression of *ERG1* and *ERG9*	β-Amyrin (**6**, 1.9 mg/L)	
*S*. *cerevisiae* BY-βA-G	Insertion of *βAS* (*G*. *glabra*), *OAS* (*M*. *truncatula*), and *AtCPR1* (*A*. *thaliana*)	OA (**1**, 71.0 mg/L), β-amyrin (**6**, 88.6 mg/L), and squalene (**4**, 141.2 mg/L)	
*S*. *cerevisiae* INVSc1	Insertion of *βAS* (*G*. *glabra*)	β-Amyrin (**6**, 4.16 mg/L)	[73]
	Insertion of *βAS* (*G*. *glabra*) and *ERG1* (*Candida albicans*)	β-Amyrin (**6**, 24.50 mg/L)	
	Insertion of *βAS* (*G*. *glabra*), *ERG1* (*C*. *albicans*), *IDI* (*Escherichia coli*) Overexpression of *ERG9* and *ERG20*	β-Amyrin (**6**, 36.50 mg/L, 75.50 mg/L *)	
	Insertion of *βAS* (*G*. *glabra*), *ERG1* (*C*. *albicans*), *IDI* (*Escherichia coli*) Overexpression of *ERG9* and *ERG20* Reconstruction of *SRE* into promoter	β-Amyrin (**6**, 85.78 mg/L, 108.60 mg/L *)	
*S*. *cerevisiae* W303-1a	Overexpression of *tHMG1* and *ERG20*	Squalene (**4**, 165.28 mg/L)	[64]
*S*. *cerevisiae* WTE	Insertion of *mix-AS* (*Catharanthus roseus*) Overexpression of *tHMG1* and *ERG20*	β-Amyrin (**6**, 1.64 mg/L), α-amyrin (**7**, 5.64 mg/L)	
	Insertion of *mix-AS* (*Catharanthus roseus*) and *ERG1* (*C*. *albicans*) Overexpression of *tHMG1*	β-Amyrin (**6**, 24.95 mg/L, 44.92 mg/L *), α-amyrin (**7**, 97.31 mg/L, 175.15 mg/L *)	
*S*. *cerevisiae* ScLCZ08	Insertion of *CrOAS* (*C*. *roseus*) and *AtCPR1* (*A*. *thaliana*)	OA (**1**, 29.49 mg/L), UA (**3**, 24.58 mg/L)	
	Insertion of *MtOAS* (*M*. *truncatula*) and *LjCPR1* (*L*. *japonicus*)	OA (**1**, 24.34 mg/L), UA (**3**, 23.37 mg/L)	
	Insertion of *CrOAS* (*C*. *roseus*) and *LjCPR1* (*L*. *japonicus*)	OA (**1**, 23.91 mg/L), UA (**3**, 22.96 mg/L)	
	Insertion of *MtOAS* (*M*. *truncatula*) and *AtCPR1* (*A*. *thaliana*)	OA (**1**, 31.41 mg/L, 155.58 mg/L *), UA (**3**, 25.85 mg/L, 123.37 mg/L *)	
*S*. *cerevisiae* JDY52	Insertion of *GgβAS* (*G*. *glabra*), *MtCYP716A12* (*M*. *truncatula*), and *AtCPR1* (*A*. *thaliana*)	OA (**1**, 2.5 ± 0.2 mg/L)	[65]
	Insertion of *GgβAS* (*G*. *glabra*), *MtCYP716A12* (*M*. *truncatula*), and *LjCPR* (*L*. *japonicus*)	OA (**1**, 6.3 ± 0.3 mg/L)	
	Insertion of *GgβAS* (*G*. *glabra*), *MtCYP716A12* (*M*. *truncatula*), and *GuCPR* (*G*. *uralensis*)	OA (**1**, 7.1 ± 0.5 mg/L)	
	Insertion of *GgβAS* (*G*. *glabra*), *MtCYP716A12*, and *MtCPR* (*M*. *truncatula*)	OA (**1**, 9.0 ± 0.7 mg/L)	
	Insertion of *GgβAS* (*G*. *glabra*), *MtCYP716A12*, and *MtCPR* (*M*. *truncatula*) Knocking out of *GAL1*	OA (**1**, 41.3 ± 3.4 mg/L)	
	Insertion of *GgβAS* (*G*. *glabra*), *MtCYP716A12*, and *MtCPR* (*M*. *truncatula*) Knocking out of *GAL1* and *GAL80*	OA (**1**, 70.3 ± 7.0 mg/L)	
	Insertion of *GgβAS* (*G*. *glabra*), *MtCYP716A12*, and *MtCPR* (*M*. *truncatula*) Knocking out of *GAL1* and *GAL80* Overexpression of *tHMG1*, *ERG1*, and *ERG9*	OA (**1**, 186.1 ± 12.4 mg/L, 606.9 ± 9.1 mg/L *)	
*S*. *cerevisiae* INVSc1	Insertion of *CYP88D6*, *CYP72A154* (*G*. *uralensis*), and *ATR1* (*A*. *thaliana*)	GA (**2**, 20.4 ± 7.7 µg/L), 11-oxo-β-amyrin (**15**, 0.5 ± 0.1 mg/L), β-amyrin (**6**, 21.23 ± 1.64 mg/L), 11α-hydroxy-β-amyrin (**16**), 30-hydroxy-11-oxo-β-amyrin (**17**), and glycyrrhetaldehyde (**18**) (trace amount)	[62]
*S*. *cerevisiae* INVSc1 (diploid)	Insertion of *CYP88D6*, *CYP72A154* (*G*. *uralensis*), and *ATR1* (*A*. *thaliana*)	GA (**2**, 31.8 ± 6.6 µg/L), 11-oxo-β-amyrin (**15**, 0.5 ± 0.06 mg/L)	
*S*. *cerevisiae* SGib	Insertion of *CYP88D6*, *CYP72A154* (*G*. *uralensis*), and *ATR1* (*A*. *thaliana*)	GA (**2**, 33.7 ± 7.4 µg/L), 11-oxo-β-amyrin (**15**, 7.5 ± 0.5 mg/L)	
	Insertion of *CYP88D6* and *ATR1* (*A*. *thaliana*)	11-oxo-β-amyrin (**15**, 3.1 ± 0.05 mg/L)	
	Insertion of *Unigene25647* and *ATR1* (*A*. *thaliana*)	11-oxo-β-amyrin (**15**, 6.0 ± 0.1 mg/L)	
	Insertion of *CYP72A63*, *Unigene25647* (*G*. *uralensis*), and *ATR1* (*A*. *thaliana*)	GA (**2**, 40.9 ± 6.4 µg/L), 11-oxo-β-amyrin (**15**, 9.16 ± 1.20 mg/L)	
	Insertion of *CYP72A154*, *Unigene25647* (*G*. *uralensis*), and *ATR1* (*A*. *thaliana*)	GA (**2**, 42.3 ± 5.8 µg/L), 11-oxo-β-amyrin (**15**, 7.22 ± 1.58 mg/L)	
	Insertion of *CYP72A154*, *Unigene25647*, and *GuCPR1* (*G*. *uralensis*)	GA (**2**, 517.4 ± 35.5 µg/L), 11-oxo-β-amyrin (**15**, 15.3 ± 1.6 mg/L)	
	Insertion of *CYP72A63*, *Unigene25647*, and *GuCPR1* (*G*. *uralensis*)	GA (**2**, 7.4 ± 1.0 mg/L, 18.9 ± 2.0 mg/L *), 11-oxo-β-amyrin (**15**, 22.6 ± 0.9 mg/L, ~80 mg/L *)	
*S*. *cerevisiae* BY-OA	Insertion of *CYP716C49* (*Crataegus pinnatifida*)	Maslinic acid (**19**, 0.06 mg/L/OD_600_)	[70]
	Insertion of *CaCYP716C49* (*Centella asiatica*)	Maslinic acid (**19**, 0.2 mg/L/OD_600_)	
	Insertion of codon-optimized *CaCYP716C49* (*C*. *asiatica*)	Maslinic acid (**19**, 0.45 mg/L/OD_600_, 384.3 mg/L *)	
	Insertion of *mix-AS* (*Eriobotrya japonica*), *VvCYP716A15*, and *CPR* (*Vitis vinifera*)	UA (**3**, 1.76 mg/L/OD_600_), OA (**2**, 0.61 mg/L/OD_600_)	
	Insertion of *mix-AS* (*E*. *japonica*), codon-optimized *CaCYP716C49* (*C*. *asiatica*), *VvCYP716A15*, and *CPR* (*V*. *vinifera*)	Corosolic acid (**20**, 0.39 mg/L/OD_600_, 141.0 mg/L*)	
*S*. *cerevisiae* BY4742	Insertion of *AaβAS* (*Artemisia annua*)	β-Amyrin (**6**, 10.8 ± 1.0 mg/L)	[68]
	Insertion of *CqβAS* (*Chenopodium quinoa*)	β-Amyrin (**6**, 10.8 ± 1.0 mg/L)	
	Insertion of *PtβAS* (*Polygala tenuifolia*)	β-Amyrin (**6**, 9.0 ± 0.7 mg/L)	
	Insertion of *LjβAS* (*L*. *japonicus*)	β-Amyrin (**6**, 8.2 ± 1.0 mg/L)	
	Insertion of *EtβAS* (*Euphorbium tirucalli*)	β-Amyrin (**6**, 8.0 ± 0.2 mg/L)	
	Insertion of *SlβAS* (*Solanum lycopersicum*)	β-Amyrin (**6**, 2.9 ± 0.3 mg/L)	
	Insertion of *CYP716AL1* (*C*. *roseus*) and *ATR2* (*A*. *thaliana*)	OA (**1**, 14.3 ± 1.6 mg/L), erythrodiol (**8**), and oleanolic aldehyde (**9**) (trace amount)	
	Insertion of *CYP716A52v2* (*P*. *ginseng*) and *ATR2* (*A*. *thaliana*)	OA (**1**, 3.0 ± 0.0 mg/L), erythrodiol (**8**), and oleanolic aldehyde (**9**) (trace amount)	
	Insertion of *SlβAS* (*S*. *lycopersicum*), *CYP716AL1* (*C*. *roseus*), and *ATR2* (*A*. *thaliana*)	OA (**1**, 3.9 ± 0.2 mg/L)	
	Insertion of *SlβAS* (*S*. *lycopersicum*), *CYP716A52v2* (*P*. *ginseng*), and *ATR2* (*A*. *thaliana*)	OA (**1**, 2.8 ± 0.0 mg/L)	
	Insertion of *AaβAS* (*A*. *annua*), *CYP716AL1* (*C*. *roseus*), and *ATR2* (*A*. *thaliana*)	OA (**1**, 8.5 ± 0.2 mg/L)	
*Yarrowia lipolytica* ATCC 201249	Insertion of *βAS* (*C*. *roseus*)	β-Amyrin (**6**, 16.0 mg/L)	[69]
	Insertion of *βAS* (*C*. *roseus*), *CYP716A12* (*M*. *truncatula*), and *ATR1* (*A*. *thaliana*)	OA (**1**, 16.3 mg/L)	
	Insertion of *βAS* (*C*. *roseus*), *CYP716A12* (*M*. *truncatula*), and *ATR1*(*A*. *thaliana*) Overexpression of *ERG1*, *ERG9*, *ERG20*, *tHMG1*	OA (**1**, 92.1 mg/L)	
	Insertion of *βAS* (*C*. *roseus*) and *CYP716A2*-linker (GSTSSG)-*t46ATR1* Overexpression of *ERG9*, *ERG20*, and *tHMG1*	OA (**1**, 129.9 mg/L, 540.7 mg/L*)	

Note: * Yield of the compound in a 5-L fermenter. ** The number in bold is the number of the compound structural formula. GA, glycyrrhetinic acid; OA, oleanolic acid; UA, ursolic acid.

**Table 3:** Biological activity of pentacyclic triterpenoid products derived by biotransformation.

Microorganism	Initial Compound, Concentration	Derivative, Yield	Biological Activity	Reference
Fungi				
*Rhizomucor miehei* CECT 2749	OA, 0.5 g/L	1β,30-dihydroxy-OA (**47**), 5.0%	- *	[108]
		7β,30-dihydroxy-OA (**48**), 6.0%	-	
		30-hydroxy-OA (**25**, queretaroic acid), 5.0%	Antitumor	
	OA methyl ester, 0.3 g/L	Methyl 3β,7β,30-trihydroxy-oleane-9(11),12-dien-28-oate (**49**), 15.0%	-	[112]
*Trichothecium roseum* (M 95.56)	OA, 0.08 g/L	7β,15α-dihydroxy-3-oxo-olean-12-en-28-oic acid (**51**), 7.5%	Anti-inflammatory	[114]
		15α-hydroxy-3-oxo-olean-12-en-28-oic acid (**52**), 6.25%	-	
*Circinella muscae* AS 3.2695	OA, 0.02 g/L	7β,15α-dihydroxy-3-oxo-olean-12-en-28-oic acid (**51**), 6.1%	Anti-inflammatory	[15]
		7β-hydroxy-OA (**53**), 5.8%	Anti-inflammatory	
		7β,21β-dihydroxy-OA (**54**), 4.2%	-	
		7α,21β-dihydroxy-OA (**55**), 3.1%	-	
		7β,15α-dihydroxy-OA (**56**), 4.9%	Anti-inflammatory	
		7β-hydroxy-3-oxo-olean-12-en-28-oic acid (**57**), 3.8%	-	
		7β,15α-dihydroxy-OA 28-*O*-β-d-glucopyranosyl ester (**58**), 4.7%	Anti-inflammatory	
		21β-hydroxy-OA 28-*O*-β-D-glucopyranosyl ester (**59**), 5.1%	Anti-inflammatory	
		OA 28-*O*-β-D-glucopyranosyl ester (**60**), 5.5%	Anti-inflammatory	
	GA, 0.06 g/L	7β-hydroxy-GA (**61**), 1.5%	Anti-inflammatory Antimicrobial	[115]
		15α-hydroxy-GA (**62**), 1.9%	Anti-inflammatory	
		7β,15α-dihydroxy-GA (**63**), 1.8%	Anti-inflammatory Antioxidant Hepatoprotective	
		3,11-dioxo-7β-hydroxy-18β-olean-12-en-30-oic acid (**64**), 1.7%	Anti-inflammatory Antimicrobial	
		7β,15α-dihydroxy-3,11-dioxo-18β-olean-12-en-30-oic acid (**65**), 2.2%	Anti-inflammatory	
		7β-hydroxy-11-oxo-18β-olean-12-en-30-oic acid 3-*O*-β-d-glucopyranoside (**66**), 1.8%	Anti-inflammatory	
		7β-hydroxy-11-oxo-18β-olean-12-en-30-oic acid 3-*O*-β-d-6′-*O*-acetyl-glucopyranoside (**67**), 1.1%	Anti-inflammatory	
		15α-hydroxy-11-oxo-18β-olean-12-en-30-oic acid 3-*O*-β-d-glucopyranoside (**68**), 2.5%	Anti-inflammatory	
		15α-hydroxy-11-oxo-18β-olean-12-en-30-oic acid 3-*O*-β-d-6′-*O*-acetyl-glucopyranoside (**69**), 1.8%	Anti-inflammatory	
		7β-hydroxy-GA 30-*O*-β-d-glucopyranoside (**70**), 2.2%	Anti-inflammatory	
*Rhizopus arrhizus* AS 3.2893	GA, 0.06 g/L	7β-hydroxy-GA (**61**), 2.8%	Anti-inflammatory Antimicrobial	[115]
		15α-hydroxy-GA (**62**), 2.2%	Anti-inflammatory	
		7β,15α-dihydroxy-GA (**63**), 1.7%	Anti-inflammatory Antioxidant Hepatoprotective	
		3β-acetoxy-7β-hydroxy-11-oxo-18β-olean-12-en-30-oic acid (**71**), 1.7%	Anti-inflammatory	
		7-oxo-GA (**72**), 1.3%	Anti-inflammatory	
		7α-hydroxy-GA (**73**), 2.6%	Anti-inflammatory	
		15α-hydroxy-7-oxo-GA (**74**), 1.3%	Anti-inflammatory	
*Colletotrichum lini* AS 3.4486	GA, 0.25 g/L	7β,15α-dihydroxy-GA (**63**), NR **	Anti-inflammatory Antioxidant Hepatoprotective	[118]
*Absidia pseudocylinderospora* ATCC 24169	GA, 1.0 g/L	7β,15α-dihydroxy-GA (**63**), 18.0%	Anti-inflammatory Antioxidant Hepatoprotective	[117]
*Cunninghamella blakesleana* CGMCC 3.970	GA, 0.1 g/L	15α,24-dihydroxy-GA (**75**), 0.11%	Anti-inflammatory	[14]
		15α,24-dihydroxy-3,11-dioxo-18β-olean-12-en-30-oic acid (**76**), 0.75%	-	
		7β,24-dihydroxy-3,11-dioxo-18β-olean-12-en-30-oic acid (**77**), 0.16%	-	
		3,11-dioxo-7β,15α,24-trihydroxy-18β-olean-12-en-30-oic acid (**78**), 0.29%	Anti-inflammatory	
		7α,24-dihydroxy-3,11-dioxo-18β-olean-12-en-30-oic acid (**79**), 1.27%	Anti-inflammatory	
*C*. *blakesleana* CGMCC 3.970	GA, 0.3 g/L	7β-hydroxy-GA (**61**), 30.0%	Anti-inflammatory Antimicrobial	[116]
		3,11-dioxo-7β-hydroxy-18β-olean-12-en-30-oic acid (**64**), 25.0%	Anti-inflammatory Antimicrobial	
*C*. *elegans* TSY-0865	GA, 0.4 g/L	7β-hydroxy-GA (**61**), 2.5%	Anti-inflammatory Antimicrobial	[119]
*Syncephalastrum racemosum* GCMCC 3.2500	UA, 0.1 g/L	7β,21β-dihydroxy-UA (**80**), 12.9%	-	[13]
		1β,21β-dihydroxy-UA (**81**), 3.9%	-	
		1β-hydroxy-21-oxo-UA (**82**), 12.1%	-	
		3β,21β-dihydroxy-urs-11-en-28,13-olide (**83**), 3.4%	Antihepatitis	
		3β,7β,21β-trihydroxy-urs-11-en-28,13-olide (**84**), 2.9%	Antihepatitis	
*Gliocladium roseum* CGMCC 3.3657	UA, 0.1 g/L	21β-hydroxy-3-oxo-urs-12-en-3,4-olide-28-oic acid (**85**), 8.0%)	-	[121]
		3,21-dioxo-urs-12-en-3,4-olide-28-oic acid (**86**), 6.25%	Antihepatitis	
		21β-hydroxy-3,4-seco-ursane-4(23),12-diene-3,28-dioic acid (**87**), 1.5%	-	
		21-oxo-3,4-seco-ursane-4(23),12-diene-3,28-dioic acid (**88**), 1.0%	Antihepatitis	
*Mucor spinosus* AS 3.3450	UA, 0.3 g/L	7β-hydroxy-UA 28-ethanone (**89**), 5.04%	Antitumor	[122]
		7β,21β-dihydroxy-UA (**81**), 1.64%	-	
		21β-hydroxy-urs-12-en-28-oic acid 3-*O*-β-d-glucopyranoside (**90**), 2.13%	-	
*Rhizopus chinensis* CICC 40335	OA, 0.2 g/L	7β,21β-dihydroxy-OA (**54**), 53.75%	-	[34]
	GA, 0.2 g/L	7β-hydroxy-GA (**61**), 77.5%	Anti-inflammatory Antimicrobial	
**Bacteria**				
*Nocardia iowensis* (DSM 45197, NRRL 5646)	OA, 0.3 g/L	Methyl 3β-hydroxy-olean-12-en-28-oat (**91**), 63.0%	Antitumor	[16]
		Methyl 3-oxo-olean-12-en-28-oat (**92**, oleanonic acid methyl ester), 5.0%	Antitumor Antiprotozoal	
		Metabolite 93 with hydroxyl and methyl groups, 2.0%	-	
*Streptomyces griseus* ATCC 13273	OA, 0.04 g/L	3β-hydroxy-olean-12-ene-28,29-dioic acid (**94**), 21.9%	-	[128]
		3β,24-dihydroxy-olean-12-ene-28,29-dioic acid (**95**), 32.7%	-	
		3β,21β,24-trihydroxy-olean-12-ene-28,29-dioic acid (**96**), 5.9%	Anti-inflammatory	
*S*. *griseus* ATCC 13273	UA, 0.04 g/L	3β-hydroxy-urs-12-ene-28,30-dioic acid (**97**), 30.4%	-	[17]
		3β,24-dihydroxy-urs-12-ene-28,30-dioic acid (**98**), 31.6%	-	
	3-oxo-UA, 0.04 g/L	3-oxo-urs-12-ene-28,30-dioic acid (**100**), 24,1%	-	
		24-hydroxy-3-oxo-urs-12-ene-28,30-dioic acid (**101**), 45.9%	-	
	2α-hydroxy-UA, 0.04 g/L	2α,3β-dihydroxy-urs-12-ene-28,30-dioic acid (**102**), 29.0%	-	
		2α,3β,24-trihydroxy-urs-12-ene-28,30-dioic acid (**103**), 15.9%	-	
*Bacillus megaterium* CGMCC 1.1741	UA, 0.2 g/L	1β,11α-dihydroxy-UA (**104**), 26.87%	-	[18]
		3-oxo-urs-12-en-28-oic acid (**99**), 6.23%	Anti-leishmania	
		1β,11α-dihydroxy-3-oxo-urs-12-en-28-oic acid (**105**), 13.5%	Anti-inflammatory	
		1β-hydroxy-3-oxo-urs-12-en-28,13-olide (**106**), 5.03%	Anti-inflammatory	
		1β,11α-dihydroxy-3-oxo-urs-12-en-28-*O*-β-d-glucopyranoside (**107**), 8.57%	-	
*B*. *subtilis* ATCC 6633	7β,21β-dihydroxy-OA, 0.2 g/L	7β,21β-dihydroxy-olean-12-en-28-oic acid 3-*O*-β-d-glucopyranoside (**108**), 46.5%	-	[34]
	GA, 0.2 g/L	GA 30-O-β-d-glucopyranoside (**111**), 27.5%	-	
	7β-hydroxy-GA, 0.2 g/L	7β-hydroxy-GA 30-*O*-β-d-glucopyranoside (**68**), 44.0%	-	
*S*. *griseus* ATCC 13273	7β,21β-dihydroxy-OA, 0.2 g/L	7β,21β,29-trihydroxy-OA (**109**), 26.0%	-	
		3β,7β,21β-trihydroxy-olean-12-ene-28,29-dioic acid (**110**), 15.0%	Neuroprotective	

Note: * Biological activity of the compound has not yet been detected. ** The value is not reported. GA, glycyrrhetinic acid; OA, oleanolic acid; UA, ursolic acid.
